# THE EFFECT OF SOCIAL SUPPORT ON THE RELATIONSHIP BETWEEN ELDER MISTREATMENT AND HEALTH IN OLDER CHINESE IMMIGRANTS

**DOI:** 10.1093/geroni/igae098.2066

**Published:** 2024-12-31

**Authors:** Soonhyung Kwon, Wendi Da, Fengyan Tang, Yanping Jiang

**Affiliations:** University of South Florida, Tampa, Florida, United States; Rutgers, the State University of New Jersey, New Brunswick, New Jersey, United States; University of Pittsburgh, Wexford, Pennsylvania, United States; Rutgers, The State University of New Jersey, New Brunswick, New Jersey, United States

## Abstract

Elder mistreatment experiences cause severe health consequences such as lower self-rated health. Despite such documented adverse effects, it is less known the potential factors that can mitigate the adverse effects of elder mistreatment on health to facilitate intervention development further. Thus, our study aims to examine the moderating role of social support from friends on the relationship between elder mistreatment and self-rated health among older Chinese immigrants. This study used five waves of PINE study involving 2,835 older Chinese immigrants aged 60 years and older. We used latent growth curve modeling (LGCM) to investigate the trajectory of self-rated health. Elder mistreatment (psychological, physical, and sexual abuse, financial exploitation, and caregiver neglect), social support from friends, and self-rated health were assessed across five waves. Our study showed social support from friends significantly moderated the adverse effects of elder mistreatment on the trajectory of self-rated health. Specifically, the detrimental effect of elder mistreatment on a faster decline in self-rated health over time was smaller at higher levels of social support from friends, showing the buffering effects of strong social support from friends on the relationship between elder mistreatment and self-reported health. Our findings suggest that transitioning friend social support from low to medium might be promising to reduce the negative effect of elder mistreatment on self-reported health among older Chinese immigrants.

